# Congenital duodenal diaphragm and enteroliths: A Unique complication

**DOI:** 10.4103/0971-9261.59609

**Published:** 2009

**Authors:** Nisar Ahmad Bhat

**Affiliations:** Department of Paediatric Surgery, Sheri Kashmir Institute of Medical Sciences, Srinagar, Kashmir, India

**Keywords:** Bile stones, duodenal diaphragm, intestinal obstruction

## Abstract

We report an unusual case of duodenal diaphragm (DD) with “wind-sock” anomaly in a 6-year-old male. The child presented with an acute duodenal obstruction precipitated by multiple pigmented stones completely blocking the duodenum.

## INTRODUCTION

Congenital duodenal obstruction (CDO) is a common cause of intestinal obstruction in the newborn. In delayed presentations, the duodenum is uninterrupted and the intrinsic obstruction is a membranous diaphragm web with a central aperture. Intra-operatively, duodenum appears normal in cases where the diaphragm has progressed on to a “wind-sock” abnormality.[1]

## CASE REPORT

A 6-year-old male child was admitted with a history of bilious vomiting of 3 days duration. The past history was that of an intermittent vomiting (both bilious and non-bilious) since the age of 1 year. The episodes of vomiting usually followed upper abdominal distention with a frequency of 2–3 times per week; also, there was poor weight gain.

**Figure 1:**
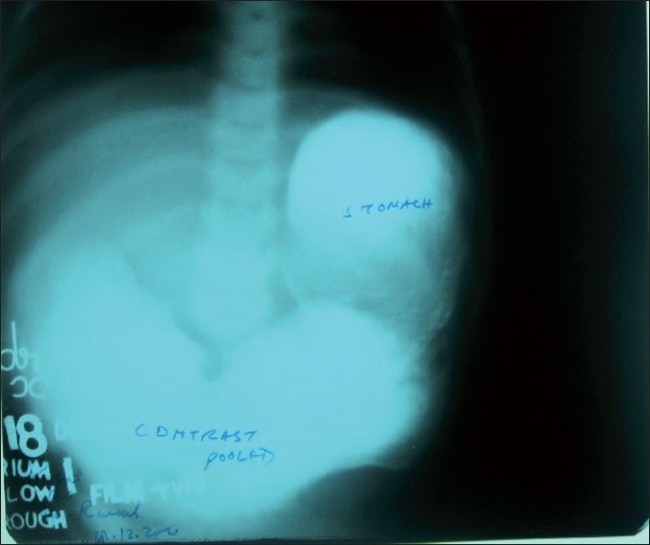
Barium enema showing hugely distended duodenum

**Figure 2:**
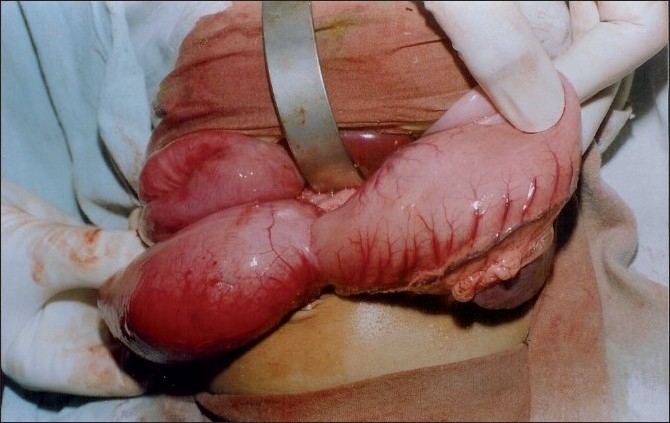
Dilated stomach with distended duodenum containing mass of stones.

**Figure 3:**
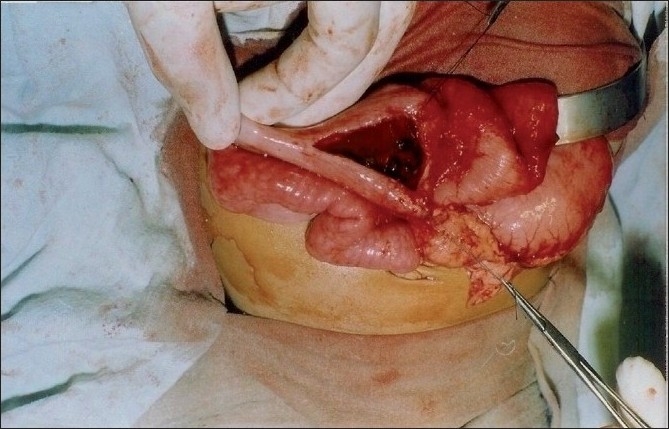
Duodenotomy shows multiple black stones.

On examination, the child was of normal intelligence but malnourished, dehydrated, and with deranged serum electrolytes. The patient was evaluated after correcting his dehydration and electrolyte imbalance. Plain x-ray of the abdomen revealed typical “double-bubble sign” with distal intestinal gas in the abdomen. Barium meal follow-through study showed both stomach and duodenum dilated and a streak of contrast going down the duodenum into the small bowel. The size of the duodenum was twice the size of the stomach [Figure 1]. Blood screening for hemolytic disorders was negative. Laparotomy showed distended stomach and hugely distended duodenum filled with a mass of stones like structures [Figure 2]. Duodenotomy revealed the mass to be multiple black stones of varying sizes and shapes [Figure 3]. After the removal of stones, wind-sock abnormality of duodenal diaphragm with a small central aperture was seen arising from the second part of the duodenum. Duodenal diaphragm was excised and duodenoplasty was done by closing the longitudinal incision in the duodenum transversely. The biliary tree and liver were normal. The postoperative period was uneventful except for *duodenal ileus* that lasted for 8 days. The patient is on regular follow-up and continues to gain weight. Chemical analysis showed stones to be of pigment variety.

## DISCUSSION

The diagnosis in CDO is usually easy and, in the majority of cases, this can be established with plain abdominal radiograph and upper gastrointestinal contrast studies.[2] The classical “double-bubble” appearance on plain abdominal radiograph is pathognomic of CDO. However, symptoms may not be specific sometimes and there may be appreciable distal gastrointestinal air in addition to double-bubble, and diagnosis in such patients may be delayed. The possibility of DD with a central hole must be considered in children with repeated attacks of vomiting even if it is non-bilious as was the case in our patient.

A variety of intrinsic and extrinsic congenital lesions may cause CDO.[3–5] It may not be possible to differentiate between them clinically or radiologically. Our patient had a classic duodenal diaphragm arising from the second part of duodenum. The weight of stones probably aggravated the process further.

Nawaz *et al.* reported a case of duodenal obstruction secondary to a duodenal diaphragm with a central hole that was obstructed by date seeds.[6] In our case, more than 50 small, black stones of varying sizes impacted the small aperture of the diaphragm causing almost complete obturation. The source of pigment stones remains mysterious as the blood investigations did not reveal any evidence of hemolytic disease in the child or the family. However, a simple progressive sedimentation of bile in a commodius duodenum with dysmotility in addition to, Ascaris lumbricoides infestation, that is endemic in the area, should be assumed as the possible reason of stone formation and accumulation.

The important lesson to learn from this child's case is that, clinically, low frequency of bilious vomiting, lack of abdominal distention, and atypical abdominal x-ray may contribute to delayed diagnosis. However, better understanding of congenital duodenal membrane behavior may prevent such delay and its sequelae like growth retardation and prolonged hospitalization.
